# Fracture propagation and permeability enhancement mechanisms induced by near-roof hydraulic fracturing in soft coal seams

**DOI:** 10.1371/journal.pone.0359936

**Published:** 2026-10-05

**Authors:** Yunheng Zhu, Xionggang Xie, Shujin Zhang, Peng Zhang, Huaiqian Liu, Deheng Jia

**Affiliations:** 1 School of Mining Engineering, Guizhou University, Guiyang, Guizhou, China; 2 Guizhou Provincial Laboratory of Intelligent Development and Efficient Utilization of Energy, Guiyang, Guizhou, China; 3 State Key Laboratory of Digital and Intelligent Technology for Unmanned Coal Mining, Anhui University of Science and Technology, Huainan, Anhui, China; Henan Polytechnic University, CHINA

## Abstract

Soft and low-permeability coal seams are prone to borehole collapse, unstable gas-extraction pathways, and a restricted stimulation range when conventional in-seam fracturing is applied. To overcome these limitations, this study proposes a near-roof hydraulic-fracturing strategy. Coal–immediate-roof assemblages from Heyuan Coal Mine were tested using a large-scale true-triaxial hydraulic-fracturing system under different triaxial in-situ stress states and injection rates. Fracture initiation, propagation, and failure modes were identified by jointly analyzing injection pressure, acoustic-emission (AE) hit counts, cumulative AE energy, and RA–AF parameters. A hydro-mechanically coupled model containing pore-pressure cohesive elements was then established, and the inferred mechanism was evaluated against field observations from the HYL1 surface L-shaped well. Increasing the in-situ stress state from 3/4/6 MPa to 9/12/18 MPa raised the breakdown pressure from 5.15 to 12.23 MPa and increased the contribution of mixed tensile–shear failure. Increasing the laboratory injection rate from 0.1 to 0.4 mL/s raised the breakdown pressure from 4.30 to 6.79 MPa, whereas the stable propagation pressure remained within 3.91–4.94 MPa. The minimum principal stress controlled the dominant fracture plane, whereas the intermediate principal stress and stress difference regulated the width and intensity of the damaged zone. After field fracturing, the mixed-gas drainage rate remained at 630–690 m^3^/d and the methane concentration exceeded 82%. These drainage responses indicate sustained fracture connectivity and improved gas-migration conditions, although permeability was not measured directly. The results support a coupled mechanism involving pore-pressure accumulation, effective-stress reduction, roof damage and pressure relief, coal–roof fracture connectivity, and improved gas migration.

## 1 Introduction

Increasing mining depth intensifies the combined effects of in-situ stress, gas pressure, and mining-induced disturbance on coal seams. Efficient gas extraction is not only critical for the prevention and control of coal mine disasters but also holds substantial significance for mitigating methane emissions from coal mines and enhancing resource utilization efficiency [1,2]. Soft and low-permeability coal seams are typically characterized by low coal matrix strength, fragmented internal structure, and poor connectivity of pore-fracture networks. When long-distance gas extraction boreholes are directly constructed within the coal seam, the combined effect of stress concentration around the borehole and gas pressure readily induces borehole wall deformation, collapse, and blockage. As the borehole gradually closes, the effective gas extraction pathways become obstructed, resulting in a rapid decay of gas extraction efficiency [2,3]. Concurrently, coal seam permeability is predominantly governed by cleat and fracture aperture, and exhibits pronounced sensitivity to effective stress and moisture saturation state. With increasing depth, elevated in-situ stress compresses primary cleats and fractures, while pore-water saturation and coal matrix deformation synergistically constrict and reconfigure gas seepage pathways—thereby intensifying the intrinsic low-permeability character of soft, structurally degraded coal seams [4,5]. Consequently, conventional coal seam drilling is inherently incapable of concurrently fulfilling the dual engineering objectives of long-distance borehole stability and large-scale permeability enhancement. A more viable strategy involves positioning horizontal wellbores within mechanically competent near-seam roof strata and applying hydraulic fracturing to propagate fractures across the coal–rock interface into the target seam. This configuration exploits the roof’s structural integrity to ensure wellbore stability, while fracture extension, induced roof damage, and attendant stress redistribution collectively enhance gas transport capacity in the underlying coal seam—thereby establishing a robust, mechanism-driven technical framework for depressurization and permeability enhancement in soft, low-permeability, structurally degraded coal seams [2,6,7].

During hydraulic fracturing, continuous fluid injection raises pressure around the wellbore. A fracture initiates when the fluid pressure overcomes the tensile resistance of the rock and the resistance to continued growth, producing nucleation at the wellbore wall or perforation tips and subsequent propagation into the far field. True-triaxial experiments show that the three-dimensional in-situ stress state governs the preferred hydraulic-fracture orientation: the minimum principal stress controls the normal to the fracture plane, whereas the horizontal principal-stress difference regulates fracture geometry; larger differences favor directional confinement and a dominant fracture path [8,9]. Injection rate modulates the balance among fluid influx, matrix imbibition, and pressure dissipation. Constant-rate, stepwise, and cyclic protocols consequently produce different propagation, injectivity, and AE responses [8,10]. Higher fluid viscosity and horizontal stress contrast can localize fracture growth and suppress secondary branching [11]. In layered coal-measure strata, fractures may cross, deflect along, become trapped at, or arrest against bedding, natural fractures, and the coal–roof interface; the outcome depends on interlayer stress contrast, elastic heterogeneity, and fracture toughness [12,13].

True triaxial hydraulic fracturing tests enable independent regulation of the three principal stress components, thereby realistically reproducing the three-dimensional non-isostatic stress state characteristic of coal–rock masses. By integrating time-resolved injection pressure data with acoustic emission (AE) response features—including hit count, cumulative energy, event location, frequency spectrum, and waveform morphology—these tests permit robust identification of fracture initiation, propagation, and coalescence stages [14,15]. Specifically, AE hit count quantifies the temporal frequency of microfracturing events; cumulative AE energy reflects the magnitude of released strain energy; and spatiotemporal patterns derived from event localization, spectral content, and waveform characteristics collectively illuminate the evolving fracture network geometry and dynamics [16,17]. Although hydraulic fracturing is predominantly governed by tensile fracture, shear- and mixed-mode fractures commonly arise due to crack deflection, slip along pre-existing weak planes (e.g., bedding or cleats), and subsequent coalescence [18]. While RA–AF parameters provide a practical means for distinguishing fracture modes, their classification thresholds are sensitive to source dimension, wave propagation path heterogeneity, and detection system triggering criteria—hence, universal threshold values cannot be reliably applied to quantify absolute mode proportions [19]. Numerical simulations complement experimental measurements by resolving otherwise inaccessible fields, such as pore pressure diffusion, stress concentration zones, progressive damage evolution, and full-field displacement distributions. The cohesive zone model (CZM) effectively captures coupled tensile–shear damage at coal–rock interfaces and reproduces cross-layer fracture propagation and deflection behaviors [20]; in contrast, the phase-field method enables fully coupled, path-independent simulation of pore pressure, displacement, and damage fields within a thermodynamically consistent framework [21]. Nevertheless, current research remains largely confined to isolated experimental characterizations, idealized homogeneous rock specimens, or direct coal-seam stimulation—lacking systematic multi-scale linkage across the integrated process chain: “fracture initiation near the roof → interlayer communication with the coal seam → roof damage-induced pressure relief → coal seam permeability enhancement → field-scale gas extraction response” [22].

To address the aforementioned research gaps, this study investigates the soft coal seam and its immediate roof strata in Heyuan Coal Mine. Large-scale coal–rock composite similar material specimens were fabricated, and true triaxial hydraulic fracturing experiments were conducted under systematically varied triaxial in-situ stress conditions and injection rates. By integrating time-resolved injection pressure data with acoustic emission (AE) metrics—including hit count, cumulative energy, and RA–AF parameters—the initiation, propagation, and tensile–shear failure modes of hydraulic fractures were robustly identified [22–24]. Building upon these experimental insights, a fluid–solid coupling model was developed, incorporating explicit representations of the coal seam, roof strata, and cohesive fracture elements. This model quantitatively elucidates how injection rate [25,26] and triaxial in-situ stress govern the evolution of damage, pore pressure, stress, and displacement fields, and reveals the mechanisms of cross-layer fracture propagation, deflection, and coalescence at the coal–rock interface [27,28]. Finally, leveraging field data from the sixth-stage hydraulic fracturing operation and post-fracturing gas drainage monitoring of the L-shaped surface well HYL1 in the 10704 working face, the experimentally observed and numerically predicted mechanisms were rigorously validated at the engineering scale. This establishes an integrated, multi-scale research framework: “true triaxial testing → AE-based fracture characterization → fluid–solid coupling simulation → field-scale validation”—thereby providing theoretically grounded and practically applicable foundations for optimizing fracturing interval selection near the immediate roof, designing operational parameters, and quantitatively evaluating pressure relief and permeability enhancement efficacy in soft, low-permeability coal seams.

## 2 Theoretical research

In coal seam roof hydraulic fracturing, high-pressure fluid induces the initiation, propagation, and interconnection of cracks within the target rock stratum. This process gradually transforms the relatively intact roof into a damaged rock mass with artificial fractures, while simultaneously modifying the load-bearing structure of the roof and the gas migration conditions in the coal seam. At the initial stage of fluid injection, the fracturing fluid primarily fills the borehole, pores, and natural fractures. The pore pressure in the vicinity of the injection end increases continuously, leading to a corresponding decrease in the effective stress of the rock matrix. Within Biot’s poroelastic framework, pore pressure affects fracture initiation and growth through its contribution to the stress carried by the solid skeleton. The corresponding effective-stress tensor for a saturated porous medium is written as [29]:


$$\begin{array}{c} \sigma^{\prime }=\sigma -\alpha pI \end{array}$$ (1)

Here, the parameters $\sigma^{\prime }$, σ, p, α and I denote the effective‑stress tensor (MPa), the total stress (MPa), the pore pressure (MPa), the Biot coefficient (dimensionless), and the second‑order unit tensor (dimensionless), respectively.

Prior to hydraulic fracturing, the intact coal seam roof exhibits high equivalent stiffness and load-bearing capacity, with overlying loads primarily transferred to the coal seam via the continuous rock mass. Post-fracturing, the generated fractures cut through and weaken the rock mass, leading to a decrease in the elastic modulus, support stiffness, and peak strength of the fractured zone. Under the equivalent continuous medium approximation, the effective elastic modulus of the fractured rock mass can be expressed as [30]:


$$\begin{array}{c} E_{\text{d}}=(\text{1}-D)E_{\text{0}} \end{array}$$ (2)

In the equation, the parameters $E_{\text{d}}$ and $E_{\text{0}}$ denote the equivalent elastic moduli of the rock mass before and after fracturing (GPa), respectively; *D* denotes the damage variable (dimensionless).

As the damage degree increases, the load carried by the fractured zone gradually decreases, and the original stress is transferred to the relatively intact rock masses on both sides, thus forming a pressure relief zone in the fractured area and a local stress concentration zone in the surrounding area. To quantitatively evaluate the change in the load transferred to the coal seam before and after fracturing, referring to the normalized definition of stress unloading degree [31], the load unloading rate is calculated by the following formula:


$$\begin{array}{c} \eta_{\text{u}}=\frac{\sigma_{\text{0}}-\sigma_{\text{1}}}{\sigma_{\text{0}}}\times \text{1}\text{0}\text{0}\% \end{array}$$ (3)

The parameters $\eta_{\text{u}}$, $\sigma_{\text{0}}$ and $\sigma_{\text{1}}$ represent the load unloading rate (%), the load transmitted to the top of the coal seam before fracturing (MPa), and the load transmitted to the top of the coal seam after fracturing (MPa), respectively. Roof hydraulic fracturing does not directly eliminate the in-situ stress of the original rock; instead, it reduces the equivalent stiffness of the fractured layer, thereby reconfiguring the transfer path of overlying loads and further mitigating the concentrated loads acting on the coal seam.

Following the formation of artificial fractures, the migration conditions of coalbed methane (CBM) are significantly improved. On one hand, these fractures can connect pre-existing cleats, bedding planes, and natural fissures, shortening the migration distance of methane from the coal matrix to extraction wells. On the other hand, the opening of fractures creates additional flow space, thereby enhancing pore-fracture connectivity and increasing the effective seepage cross-sectional area. For fractures that are approximately smooth, parallel, and satisfy laminar flow conditions, their equivalent permeability and volumetric flow rate can be expressed in accordance with the cubic law for fractures [32,33]:


$$\begin{array}{c} k_{\text{f}}=\frac{b^{\text{2}}}{\text{1}\text{2}} \end{array}$$ (4)

$$\begin{array}{c} Q_{\text{f}}=\frac{wb^{\text{3}}}{\text{1}\text{2}\mu L}\nabla p \end{array}$$ (5)

In Eqs. (4) and (5), $k_{\text{f}}$ is the fracture equivalent permeability (m²); b is the fracture aperture (m). $Q_{\text{f}}$ is the fracture volumetric flow rate (m^3^/s); w, b, and *L* are the fracture width, aperture, and flow path length (m), respectively, in meters; *μ* is the fluid dynamic viscosity (Pa·s); ∇p is the pressure difference across the fracture ends (Pa).

The field engineering results exhibit good consistency with the aforementioned mechanism. The on-site gas extraction performance is evaluated based on the mixed gas flow rate and methane volume fraction, and the pure methane extraction rate is converted using the following formula:


$$\begin{array}{c} Q_{\text{C}{\text{H}}_{\text{4}}}=Q_{\text{mix}}\frac{C_{\text{C}{\text{H}}_{\text{4}}}}{\text{1}\text{0}\text{0}} \end{array}$$ (6)

The pure methane extraction rate is denoted by $Q_{{\text{CH}}_{\text{4}}}\left({\text{m}}^{\text{3}}/\text{d}\right)$, while the mixed gas extraction rate is denoted by $Q_{\text{mix}}$ (m^3^/d), and the methane volume fraction is denoted by $C_{{\text{CH}}_{\text{4}}}$ (%).

## 3 Experimental study

### 3.1 Raw coal-rock sample testing and similar material specimen preparation

The raw coal and roof rock samples were collected from Heyuan Coal Mine. The original blocks were drilled, cut, and polished to prepare cylindrical, disc-shaped, and wedge-shaped shear specimens, which were then subjected to uniaxial compression tests, Brazilian splitting tensile strength tests, and wedge shear strength tests. The experimental procedure is illustrated in Fig 1. The average uniaxial compressive strengths (UCS) of the natural roof rock and raw coal were determined to be 67.68 MPa and 11.00 MPa, respectively, while their average tensile strengths (obtained via Brazilian splitting test) were 13.38 MPa and 4.37 MPa, respectively. These experimentally measured mechanical parameters were employed to define the strength grade of the similar materials.

**Fig 1:**
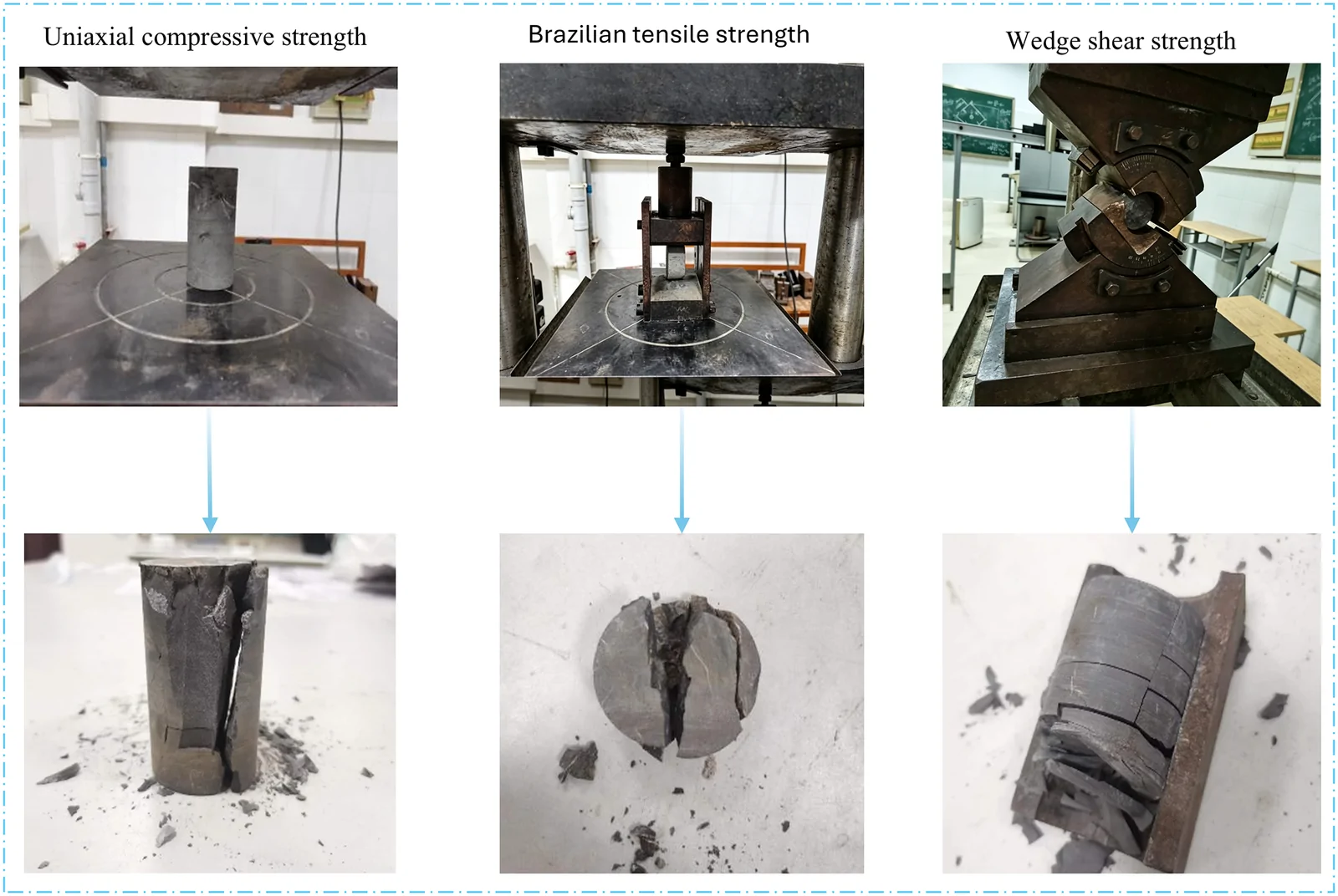
Loading apparatus for three types of coal-rock mechanical tests and specimen failure modes.

The physical simulation specimen adopts a coal-rock composite structure. The coal briquettes were formulated using coal powder, cement, and gypsum as raw materials, whereas the roof simulant material was composed of sand, cement, and gypsum. After screening multiple mix‑proportion schemes, the optimal formulation for coal briquettes was determined as gypsum: cement: coal powder = 1:4:6, and the optimal mix ratio for the roof simulant material was sand: cement: gypsum = 3:4:1. The corresponding uniaxial compressive strengths (UCS) of the two materials were 1.168 MPa and 7.36 MPa, respectively. Their tensile strengths, determined by the Brazilian splitting test, were 0.3924 MPa and 1.157 MPa, respectively.

The test specimens measured 200 mm × 200 mm × 200 mm and were cast in layers following the coal-seam and roof sequence. After each layer was poured, mechanical vibration and tamping expelled entrapped air. Specimens were kept at room temperature for 72 h before demolding and then cured for 10 d. During casting, a steel pipe was embedded at the center to reserve the fracturing borehole and removed after sufficient strength developed. After the borehole wall had dried, the injection pipe was installed and sealed with a high-strength adhesive; the assembly was then left undisturbed for approximately 60 h to ensure pressure resistance and sealing. The complete specimen-preparation workflow is shown in Fig 2.

**Fig 2:**
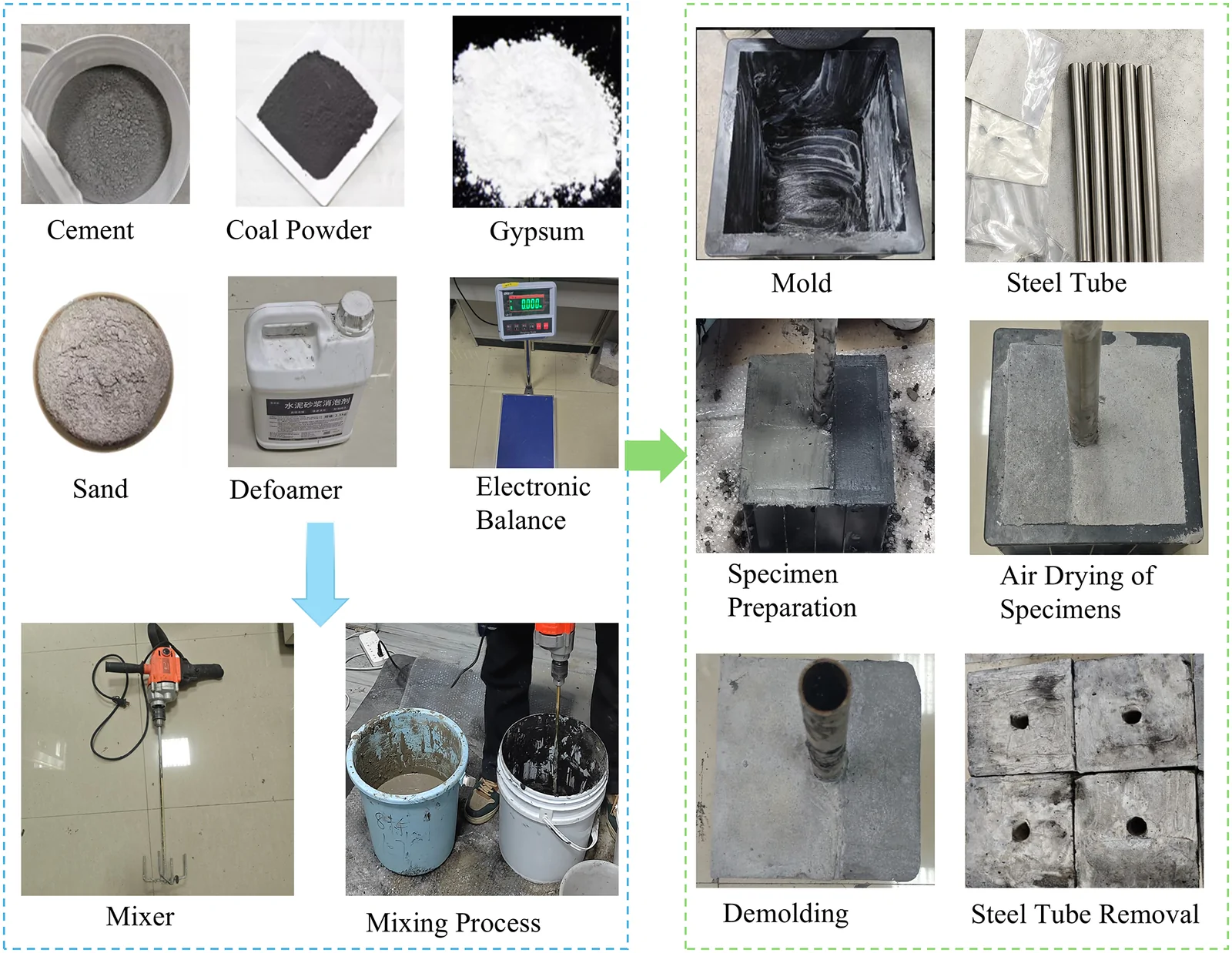
Specimen-preparation workflow.

### 3.2 Experimental design and implementation method

Laboratory hydraulic-fracturing tests provide a controlled means of examining fracture initiation, propagation, and connectivity in the roof of a soft coal seam. Field-scale fracture evolution depends on rock properties, natural discontinuities, and the in-situ stress field [34], as well as on operational parameters such as injection rate, injection pressure, and injection mode. We therefore used a control-variable design to examine in-situ stress level and injection rate. Six listed groups were used, including one shared reference experiment, as detailed in Table 1.

**Table 1:** Hydraulic-fracturing test conditions under different controlling factors.

No.	Investigated factor	Injection rate/(mL·s^-1^)	In‑situ stress $\left(\sigma_{X},\sigma_{Y},\sigma_{Z}\right)$/MPa	Fracturing fluid
1	Injection rate	0.1	3/4/6	Clear water with tracer
2	Injection rate	0.2	3/4/6	Clear water with tracer
3	Injection rate	0.4	3/4/6	Clear water with tracer
4	In‑situ stress level	0.2	3/4/6	Clear water with tracer
5	In‑situ stress level	0.2	6/8/12	Clear water with tracer
6	In‑situ stress level	0.2	9/12/18	Clear water with tracer

Note: Groups 2 and 4 refer to the same experiment (injection rate, 0.2 mL/s; in-situ stress, 3/4/6 MPa), which served as the common reference condition for the injection-rate and in-situ-stress comparisons.

Four functional units formed the experimental platform: a large-scale true-triaxial loading system, a hydraulic-fracturing injection module, an AE monitoring system, and a synchronized multiparameter acquisition system. It enables the simulation of the triaxial stress state of specimens, constant-flow fluid injection loading, real-time monitoring of crack evolution processes, and synchronous acquisition of multi-physical field parameters including water pressure, flow rate, load, and displacement [35], as illustrated in Fig 3:

**Fig 3:**
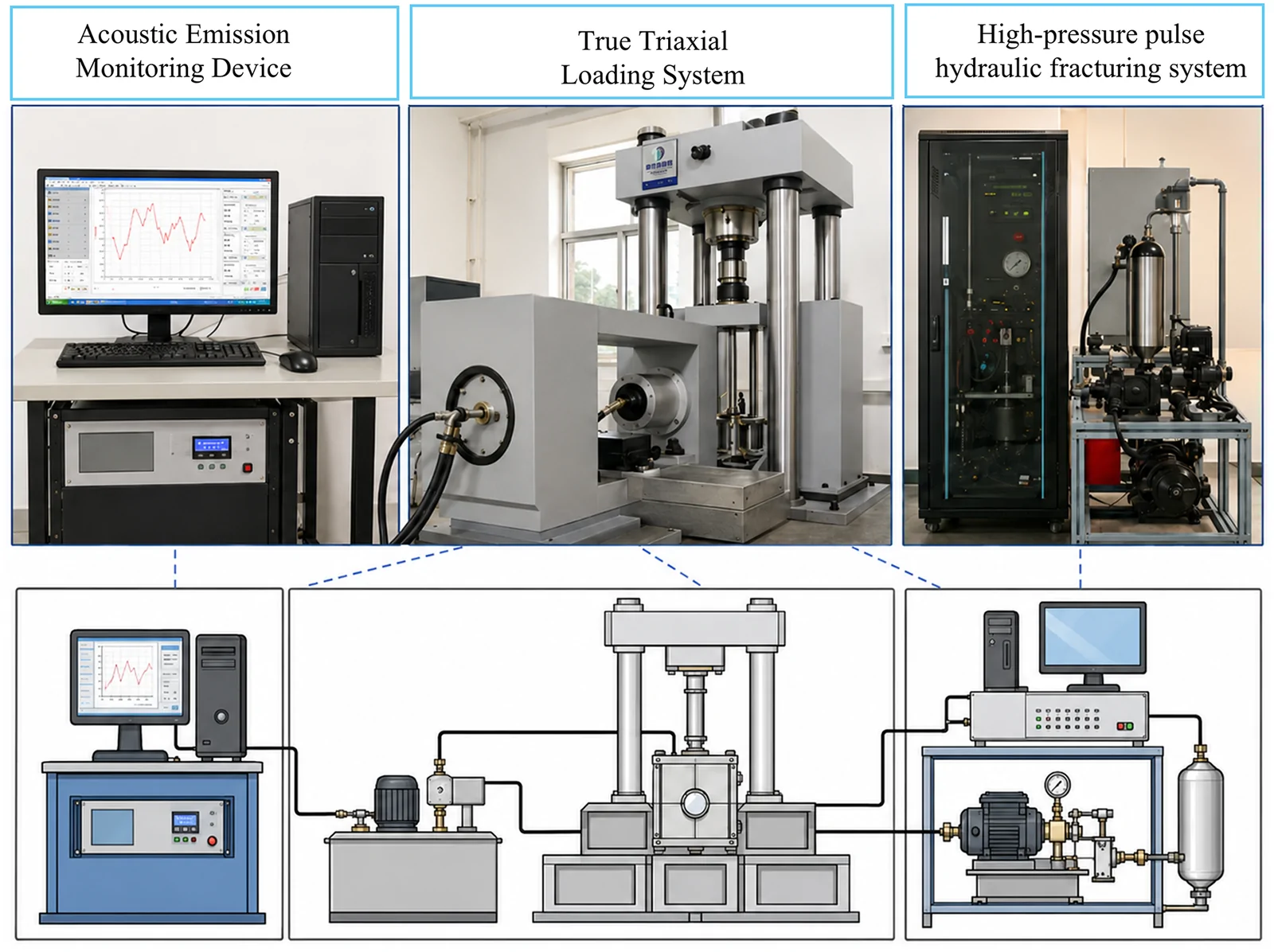
Hydraulic-fracturing test system.

Before testing, acoustic-wave velocity was calibrated by pencil-lead break tests, after which the specimen, pipelines, and AE channels were checked. AE signals were acquired synchronously using an eight-channel DS5-16B system and eight RS-14A sensors arranged on two opposite specimen surfaces. Each sensor had an operating frequency range of 20–90 kHz and a nominal center frequency of 50 kHz. The sampling frequency was 3 MHz per channel, the preamplifier gain was 40 dB, and threshold triggering was used. RA was defined as rise time divided by peak amplitude, whereas AF was defined as AE counts divided by signal duration. Accordingly, the RA–AF distribution was interpreted qualitatively rather than used to assign absolute tensile- and shear-fracture proportions. Triaxial stress was then applied along the prescribed path and held for 10 min after reaching the target. Fluid was injected at a constant rate until leakage or a sudden pressure drop indicated a connected fracture. Fracture initiation, propagation, and connectivity were evaluated from the pressure curve, tracer distribution, and AE response.

### 3.3 Experimental results and analysis

#### 3.3.1 Effect of injection rate on hydraulic-fracturing pressure response.

As shown in Fig 4, under the 3/4/6 MPa triaxial stress state, the pressure response varied markedly with injection rate. Increasing the rate from 0.1 to 0.4 mL/s raised the breakdown pressure from 4.2968 to 6.7859 MPa, an increase of approximately 57.9%. Similar rate-related increases in breakdown pressure have been reported for coal and crystalline rock [23,36]. At higher rates, fluid enters the pore–fracture system faster. When supply exceeds matrix permeation and leak-off, pressure accumulates more rapidly around the injection hole and fracture tips; at lower rates, permeation and leak-off consume a larger fraction of the injected fluid and delay pressure buildup [36,37]. After stable propagation begins, however, the stable propagation pressure remains within 3.91–4.94 MPa and varies less than the breakdown pressure.

**Fig 4:**
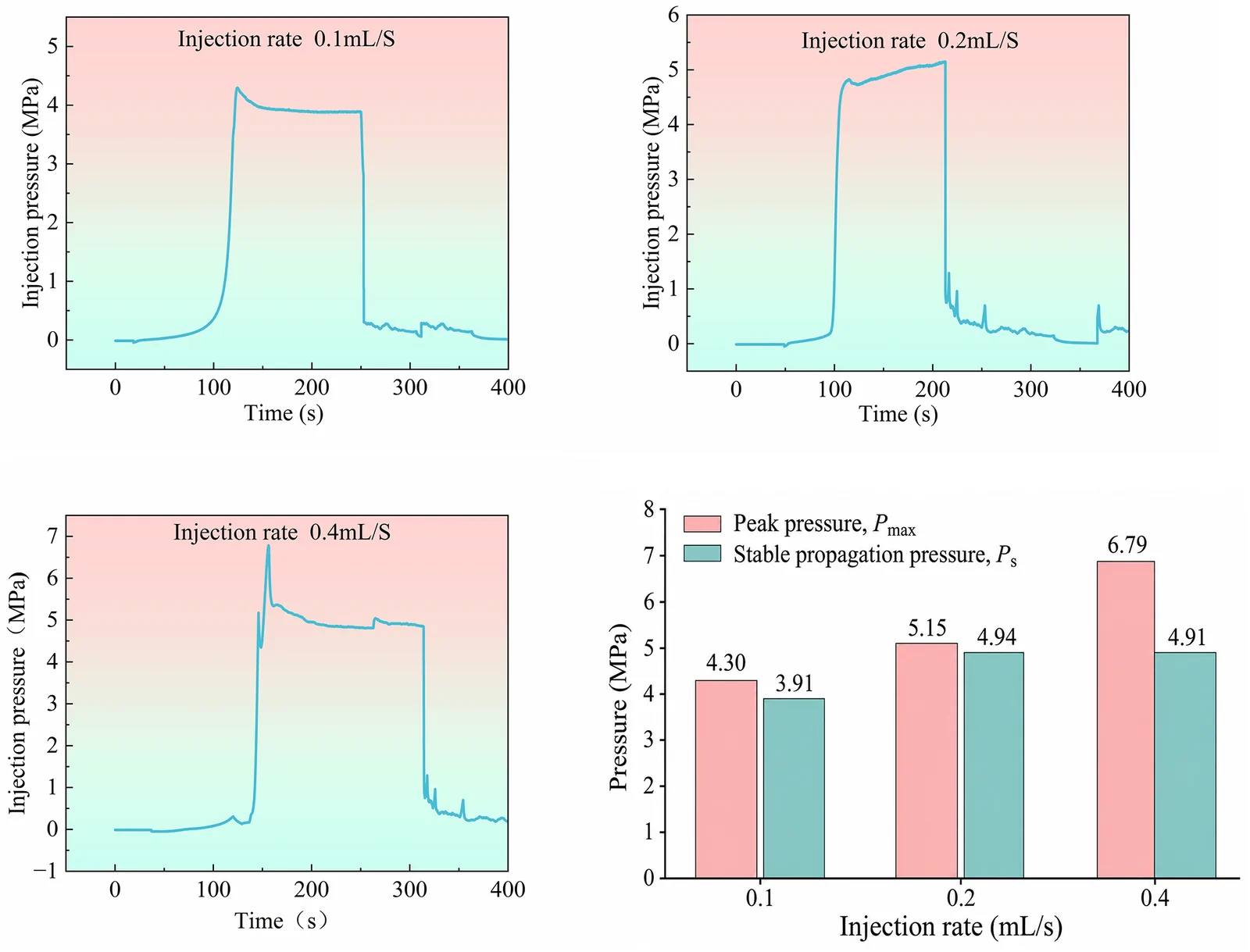
Pressure responses and characteristic pressures at different injection rates.

Once a continuous flow path forms, propagation is governed by fracture geometry, fluid supply, leak-off, and confining stress. Within the tested laboratory conditions, injection rate therefore has a stronger influence on breakdown pressure than on stable propagation pressure. This interpretation is limited to the present specimen geometry, material properties, boundary conditions, and pressure-measurement method.

#### 3.3.2 Influence of in-situ stress on hydraulic-fracturing pressure response.

To isolate the effect of the in-situ stress level on hydraulic fracture initiation and propagation in coal seams, three true-triaxial hydraulic fracturing tests were [38] conducted under stress states of 3/4/6, 6/8/12, and 9/12/18 MPa, while the injection rate was maintained at 0.2 mL/s. These test conditions correspond to Groups 4–6 in Table 1. The stress-dependent responses of the specimens were evaluated by jointly comparing their injection-pressure histories, post-fracturing fracture morphologies, and acoustic emission characteristics. The pressure and AE histories are summarized in Fig 5, and the corresponding RA–AF distributions are shown in Fig 6. This comparative design enabled the effects of increasing in-situ stress on fracture initiation and subsequent propagation to be identified [12,39].

**Fig 5:**
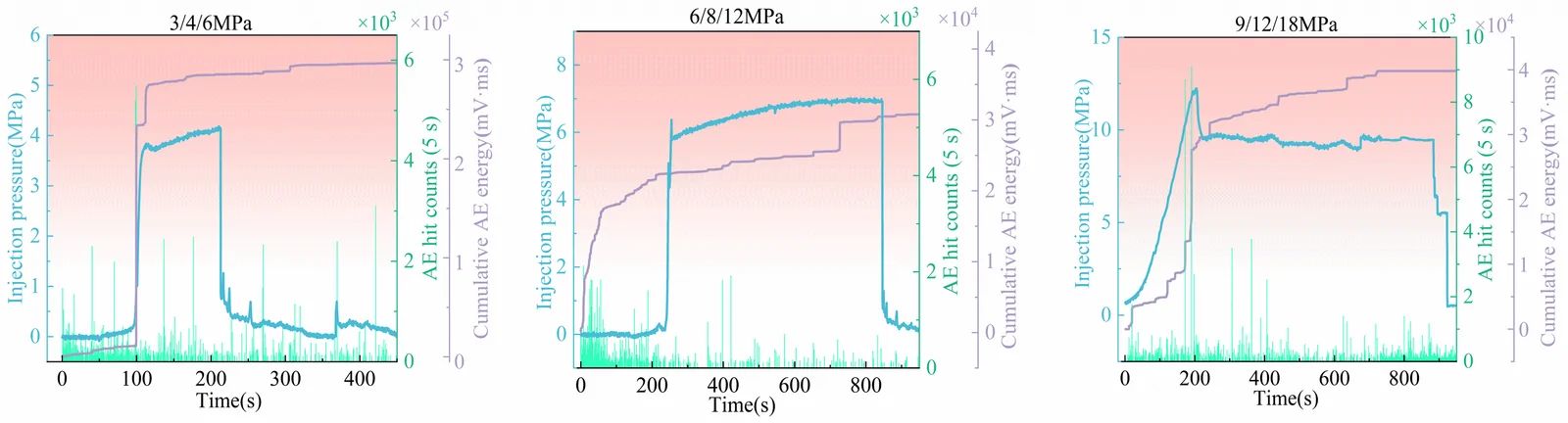
Temporal evolution of injection pressure, AE hit counts, and cumulative AE energy.

**Fig 6:**
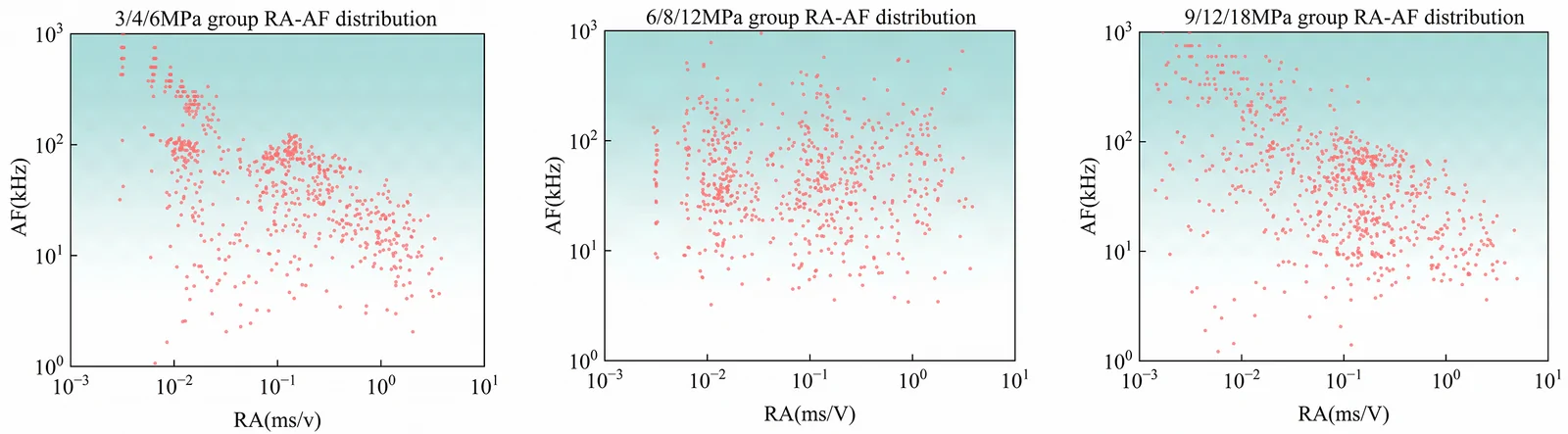
RA–AF distributions.

With all other experimental variables held constant, increasing the triaxial in-situ stress state from 3/4/6 MPa to 6/8/12 MPa and subsequently to 9/12/18 MPa resulted in an overall rise in both the peak injection pressure and stable propagation pressure. The elevated stress level intensified the normal compressive confinement acting across the fracture surfaces and promoted the closure of pre-existing fractures. Consequently, greater fluid pressure was required to initiate, open, and sustain hydraulic fracture propagation. Similar laboratory studies have demonstrated that confining stress affects the pressure required for hydraulic fracture development, while pre-existing fracture networks strongly influence pressure evolution and fracture behavior in coal [39].

Under the 3/4/6 MPa stress condition, the relatively low confinement facilitated the activation of weak structural planes and pre-existing fractures within the coal mass. AE hit counts occurred frequently but were comparatively dispersed, whereas cumulative AE energy increased sharply during fracture initiation. Most RA–AF data points occupied the low-RA and high-AF region, indicating that tensile cracking was the predominant failure mode. When considered together with the observed fracture morphology, this AE distribution is consistent with branched fracture development.

At 6/8/12 MPa, the prolonged high-pressure stage was accompanied by stepwise increases in AE hit counts and cumulative AE energy. This response reflects repeated cycles of crack initiation, temporary arrest, renewed pressure accumulation, and subsequent re-propagation. Meanwhile, the wider RA–AF distribution suggests an increasing contribution from shear cracking, indicating a gradual transition from tensile-dominated failure to a mixed tensile–shear mechanism. AE count, energy, and crack-classification parameters have similarly been used to identify hydraulic fracture development stages and distinguish tensile and shear cracking in laboratory fracturing test [16].

When the in-situ stress increased to 9/12/18 MPa, stronger fracture closure and propagation resistance suppressed deflection and branching. AE activity became more stage-specific and localized, and cumulative AE energy increased stepwise, indicating a shift from dispersed, low-energy microcracking toward fewer localized high-energy events. The RA–AF distribution expanded toward higher RA and lower AF, consistent with greater contributions from frictional sliding, shear cracking, and mixed tensile–shear failure. Thus, increasing in-situ stress shifted the response from tensile-dominated branching under low confinement toward localized, high-pressure mixed-mode failure.

## 4 Numerical simulation

### 4.1 Model and simulation scheme

A 20 m × 20 m × 20 m three-dimensional coal–roof model was established in Abaqus. The vertical coordinate is y; the coal occupies y = 0–8 m and the roof occupies y = 8–20 m. The injection point is located at y = 10 m, 2 m above the coal–roof interface. The coal has an elastic modulus of 6.0 GPa, Poisson's ratio of 0.36, and hydraulic-conductivity input of 1.0 × 10^-8^ m/s. The corresponding roof values are 18.0 GPa, 0.31, and 1.0 × 10^-7^ m/s. Pore-pressure cohesive elements represent damage evolution, fracture flow, and leak-off into the porous medium [40,41]. The pore fluid had a dynamic viscosity of 0.001 Pa·s. The model geometry and coal–roof arrangement are shown in Fig 7. Two simulation groups were defined:

**Fig 7:**
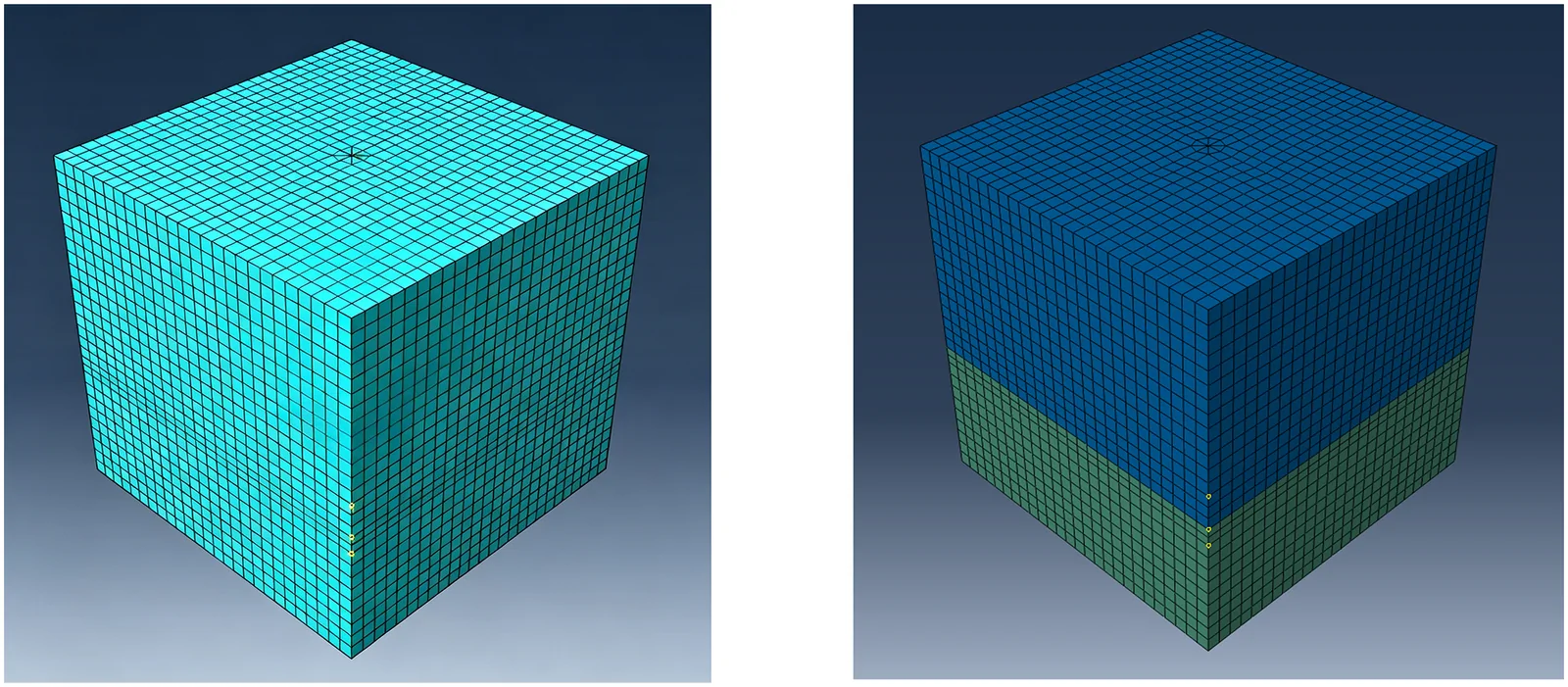
Numerical model and prescribed cohesive-element network for near-roof hydraulic fracturing. (a) Overall model (b) Coal-rock interface model.

Group 1: The in-situ stress was fixed at 6/4/10 MPa, and four constant-rate simulations were conducted at 0.4, 0.6, 0.8, and 1.0 mL/s.
Group 2: The injection rate was fixed at 0.6 mL/s, and the triaxial stress combinations were varied to compare fracture propagation when either the maximum horizontal stress or the vertical stress was dominant.

The injection-rate ranges were selected independently for the three research scales: 0.1–0.4 mL/s for the 0.2 m laboratory specimens, 0.4–1.0 mL/s for the 20 m numerical model, and 5.0–9.0 m^3^/min for field operations. These ranges reflect different geometries, boundary constraints, fluid-loss environments, and operational objectives; they are not assumed to be dynamically similar and are not directly scaled. The cross-scale comparison is therefore restricted to qualitative response trends and mechanistic consistency.

### 4.2 Boundary conditions and fracture criteria

Mechanical loading was prescribed through a mixed traction–displacement boundary scheme, while all external hydraulic boundaries except the injection point were impermeable [42]. A constant volumetric flow rate was applied at the injection point, and deformation, pore-pressure evolution, and progressive damage were solved within a coupled framework [43]. Eight-node pore-pressure cohesive elements (COH3D8P) were inserted only on a prescribed admissible fracture network, not throughout the three-dimensional bulk. In the full 20 m model, the active network contained 732 cohesive elements: 692 zero-geometric-thickness elements formed a horizontal candidate plane at y = 7 m (1 m below the coal–roof interface at y = 8 m), and 40 elements formed four short vertical strips around the injection axis from y = 7–10 m, crossing the coal–roof interface and linking the roof injection point to the coal-side plane. All active cohesive elements used the same Mid-Cohe material law. The coupled solution determines where damage initiates, evolves, or arrests on this network, but it cannot create a crack outside the inserted surfaces. No independent mesh-refinement or alternative-path study was available; therefore, we do not claim that the absolute trajectory is mesh- or path-independent. The numerical results are used only to compare pressure, stress, displacement, and damage activation on an identically discretized candidate network.

The cohesive elements used a specified constitutive thickness of 0.001 m. The uncoupled normal and two tangential traction–nominal-strain stiffnesses were E_nn = E_ss = E_tt = 2.0 × 10^10^ Pa; with the specified thickness, these correspond to initial traction–separation slopes of 2.0 × 10^13^ Pa/m. Damage initiated when the maximum nominal stress criterion max{<t_n > /t_n0, t_s/t_s0, t_t/t_t0} reached 1, where compression does not contribute through the Macaulay bracket and t_n0 = t_s0 = t_t0 = 0.8 MPa. Linear, energy-based damage evolution used G_Ic = G_IIc = G_IIIc = 4.0 kJ/m^2^ and the Benzeggagh–Kenane mixed-mode law with exponent 2.0. A cohesive-section viscosity control of 0.01 was used. Hydraulic transport within the opened gap used a dynamic viscosity of 0.001 Pa·s, and the top and bottom leak-off coefficients were each 1.0 × 10^-13^ m/(Pa·s); no independent constant interface permeability was assigned. The same law was assigned to the cohesive strips crossing the coal–roof boundary. Accordingly, damage can cross that boundary only along the vertical inserted strips, can turn into the horizontal candidate plane only at their prescribed junction, and arrests where the stress-initiation or mixed-mode energy condition is not met. The model does not resolve competition with an arbitrary deflected path outside this network. SDEG varies from 0 for an intact interface to 1 for complete failure.

The injection-rate series used a 300 s coupled consolidation step with automatic time incrementation, an initial increment of 0.01 s, a minimum increment of 10^-10^ s, a maximum increment of 10 s, and no more than 10,000 increments. The prescribed rate was ramped linearly from zero to its target during the first 10 s and was then held constant. Integrating these nominal total-rate histories gives injected volumes of 118, 177, 236, and 295 mL for target rates of 0.4, 0.6, 0.8, and 1.0 mL/s, respectively. Thus, the simulations are time-matched rather than volume-matched: injection rate and cumulative injected volume increase together, and the results should be interpreted as the combined response to injection intensity and fluid supply within a fixed simulation window, not as an isolated causal effect of rate.

### 4.3 Numerical simulation results and analysis

#### 4.3.1 Impact of high liquid-injection rate.

To examine the fixed-duration response to numerical injection intensity, simulations were conducted at 0.4, 0.6, 0.8, and 1.0 mL/s under an in-situ stress state of 6/4/10 MPa. The 300 s simulation window was identical in all cases; consequently, the cumulative injected volume increased with the prescribed rate, as specified in Section 4.2. The injection-rate cases are summarized in Table 2.

**Table 2:** Numerical simulation schemes at different injection rates.

No.	Injection rate	Stress components (MPa)			Fracturing mode
	(mL/s)	σx (σH)	σz (σh)	σy (σv)	
1	0.4	6	4	10	Constant‑rate flow
2	0.6	6	4	10	Constant‑rate flow
3	0.8	6	4	10	Constant‑rate flow
4	1.0	6	4	10	Constant‑rate flow

Fig 8 shows the coupled damage, pore-pressure, equivalent-stress, and displacement responses at different numerical injection rates. At 0.4 mL/s, activation of the prescribed cohesive network remained near the injection point and the pressure, stress, and displacement disturbances were localized. Within the fixed 300 s window, this case also had the smallest nominal injected volume (118 mL).

**Fig 8:**
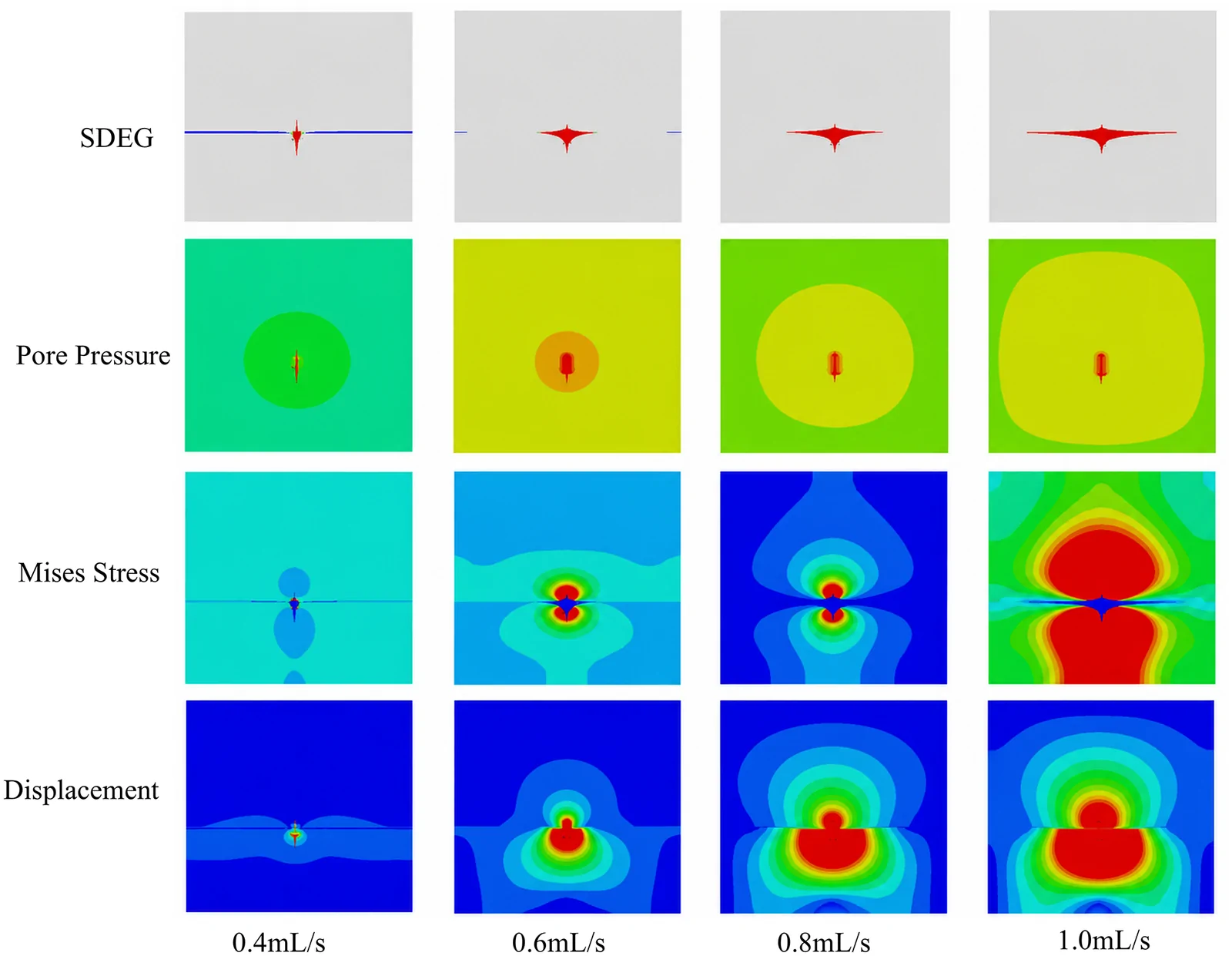
Coupled fracturing responses at different numerical injection rates.

At 0.8 and 1.0 mL/s, the high-SDEG region extended farther along the prescribed cohesive surfaces, the pore-pressure disturbance expanded, and stronger equivalent-stress and displacement responses developed around the activated elements. Under the common 300 s duration, these cases received larger nominal fluid volumes (236 and 295 mL), so the enhanced response reflects the combined increase in injection intensity and cumulative fluid supply. Comparable rate-dependent increases in pressure and fracture development have been reported in numerical and true-triaxial studies [23,44].

The 0.8 and 1.0 mL/s cases activated broadly similar branches of the prescribed cohesive network, although the latter showed stronger damage, stress, and displacement concentrations. Thus, within the fixed-duration design, increasing fluid supply intensified damage along the admissible surfaces more than it changed which branch was activated. This result must not be interpreted as an unconstrained prediction of fracture orientation; the available directions were fixed by the inserted cohesive topology. Within that constraint, the response remains consistent with the tendency for hydraulic fractures to develop perpendicular to the minimum principal stress [45].

Overall, larger prescribed rates produced faster pressure buildup and a broader coupled disturbance within the 300 s window. Because total injected volume increased with rate, these simulations do not isolate a rate-only effect. The results should be interpreted as a comparison of fixed-duration injection strategies; an equal-volume simulation series would be required to separate rate from cumulative-volume effects.

#### 4.3.2 Impact of in-situ stress.

To compare hydraulic-fracture propagation under different differential-stress conditions, material parameters, boundary conditions, the injection method, and the injection rate were held constant, with the rate fixed at 0.6 mL/s. In the numerical geometry, y is vertical and x and z are horizontal. The tabulated order is therefore (σx/σz/σy) = (σH/σh/σv): 6/3/8, 6/3/10, 8/5/3, and 8/6/3 MPa. For the first two states, σz  =  σh  =  3 MPa is the minimum principal stress while σy  =  σv increases from 8 to 10 MPa; for the last two states, σy  =  σv  =  3 MPa is the minimum principal stress. The stress cases are summarized in Table 3.

**Table 3:** Numerical simulation schemes under different in-situ stress states.

No.	Injection rate	Stress components (MPa)			Fracturing mode
	(mL/s)	σx (σH)	σz (σh)	σy (σv)	
1	0.6	6	3	8	Constant‑rate flow
2	0.6	6	3	10	Constant‑rate flow
3	0.6	8	5	3	Constant‑rate flow
4	0.6	8	6	3	Constant‑rate flow

Fig 9 shows that stress configuration affected damage activation within the prescribed cohesive network. For 6/3/8 and 6/3/10 MPa, σz = σh = 3 MPa is the minimum principal stress, while σy = σv increases from 8 to 10 MPa. In both cases, the high-SDEG zone remained concentrated around the injection point. Increasing σv intensified local stress and displacement but produced little expansion of the damaged region, indicating that stronger normal confinement increased resistance to opening and redirected more injected energy toward near-wellbore pressurization and localized stiffness degradation.

**Fig 9:**
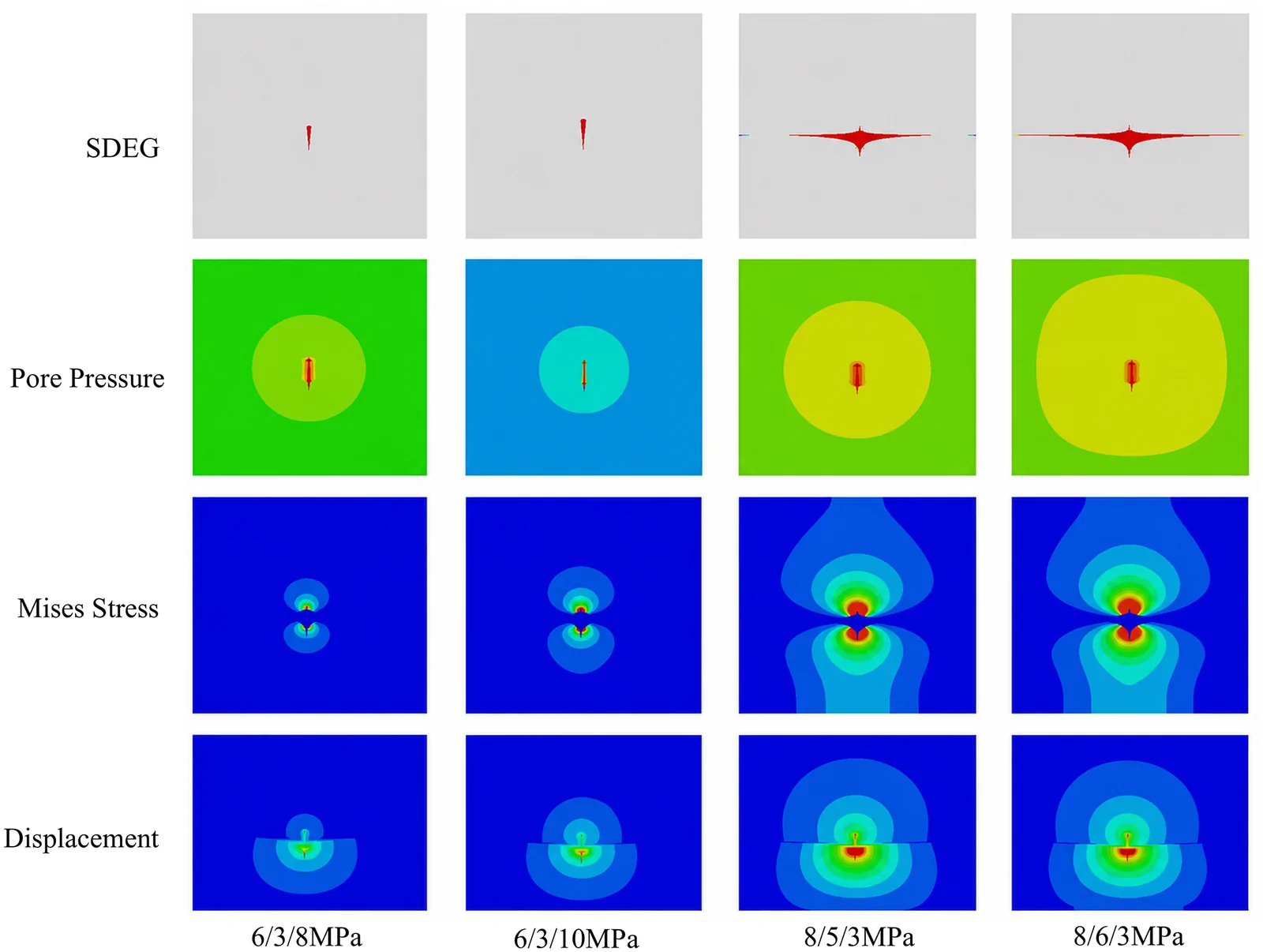
Fracturing-response contours under different in-situ stress states.

For 8/5/3 and 8/6/3 MPa, σy = σv = 3 MPa is the minimum principal stress, whereas σx = σH = 8 MPa and σz = σh = 5 or 6 MPa. The horizontal branch of the prescribed cohesive network consequently showed greater SDEG activation as the minimum-stress direction changed. This is evidence of stress-dependent branch activation within the predefined network, not proof of an unconstrained reorientation. Hydraulic fractures generally develop approximately perpendicular to the minimum principal stress, although heterogeneity and stress differences can modify the path [46,47]. The associated pressure, stress, and displacement disturbances also occupied a broader area than in the 6/3/8 and 6/3/10 MPa cases.

A comparison between the 8/5/3 and 8/6/3 MPa cases shows that the maximum–minimum principal-stress difference was unchanged while the intermediate principal stress increased from 5 to 6 MPa. The same prescribed horizontal branch was activated in both cases, although the pore-pressure diffusion, local stress concentration, displacement, and SDEG magnitude differed. Within the imposed cohesive topology, the minimum-stress direction therefore affects relative branch activation, whereas the intermediate stress modulates the local hydro-mechanical response. This comparison cannot establish the trajectory of a freely propagating crack. Previous true-triaxial research has likewise shown that the intermediate principal stress can influence fracture reactivation resistance, deformation, and failure mode [48].

## 5 Engineering verification

### 5.1 Engineering geology and L-shaped well layout

To evaluate the field-scale applicability of the mechanisms and trends identified through the laboratory true-triaxial tests and numerical simulations, a staged hydraulic fracturing trial was conducted using a surface L-shaped horizontal well at the 10704 working face of Heyuan Coal Mine. The target No. 7 coal seam has an average thickness of approximately 2.33 m and consists predominantly of fragmented and pulverized coal, resulting in a loose structure and poor overall integrity. Consequently, completing a long horizontal section directly within the coal seam would involve a high risk of borehole collapse, coal-fines migration, and wellbore instability. To overcome these engineering limitations, the horizontal section of Well HYL1 was positioned in the relatively competent near-roof strata, approximately 2 m above the roof of the No. 7 coal seam. This comparatively intact rock interval provided a stable host for the wellbore and served as the fracture-initiation horizon, from which the induced fractures could propagate downward and connect with the underlying loose coal seam. The working-face layout and the near-roof trajectory of Well HYL1 are shown in Fig 10.

**Fig 10:**
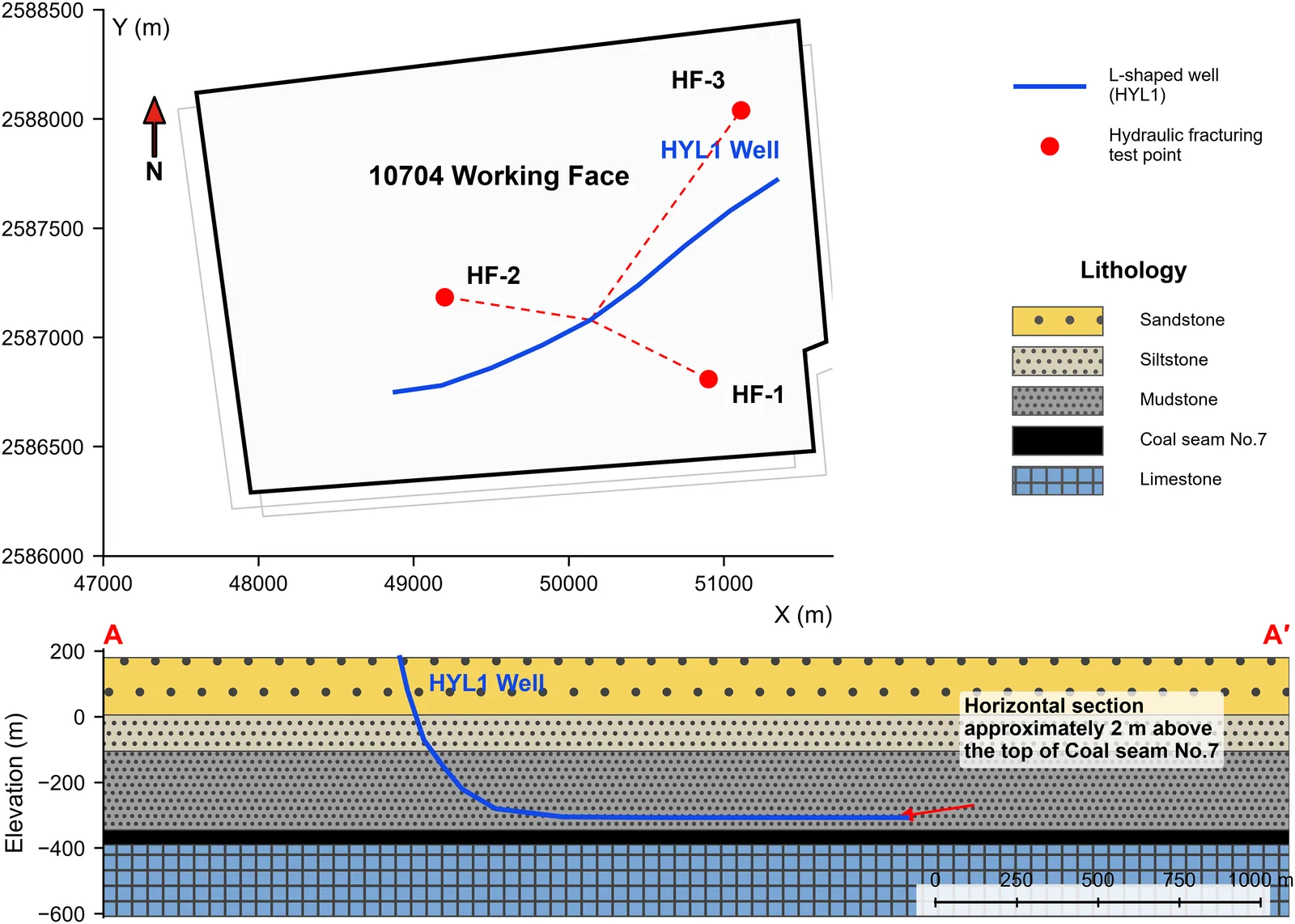
Layout of Working Face 10704 and Well HYL1. The figure was independently drawn by the authors from field measurements and operational data; no third-party map, drawing, or satellite image was reproduced.

Hydraulic-fracturing measurements yielded maximum and minimum horizontal principal stresses of 2.56 and 1.87 MPa, respectively, while the overburden calculation gave a vertical stress of 4.375 MPa. The azimuth of the maximum horizontal principal stress is approximately 348° (clockwise from true north, with 0° corresponding to true north), which corresponds to a direction close to N12°W. The in-situ stress magnitude relationship in the study area is characterized by $\sigma_{v}>\sigma_{H}>\sigma_{h}$, indicating a normal faulting stress regime dominated by vertical stress. Under this stress regime, hydraulic fractures generally tend to form fracture planes with their normals perpendicular to the minimum horizontal principal stress ($\sigma_{h}$) and strike directions approximately parallel to the maximum horizontal principal stress ($\sigma_{H}$). However, at the engineering scale, fracture propagation paths are collectively influenced by bedding planes, natural fractures, coal-rock interfaces, and local lithological heterogeneities. Therefore, in-situ stress primarily dictates the overall orientation of fracture propagation, while local structural planes further govern fracture deflection, branching, and interlayer penetration behaviors. The in-situ stress test, borehole observations, and interpreted fracture orientation are summarized in Fig 11.

**Fig 11:**
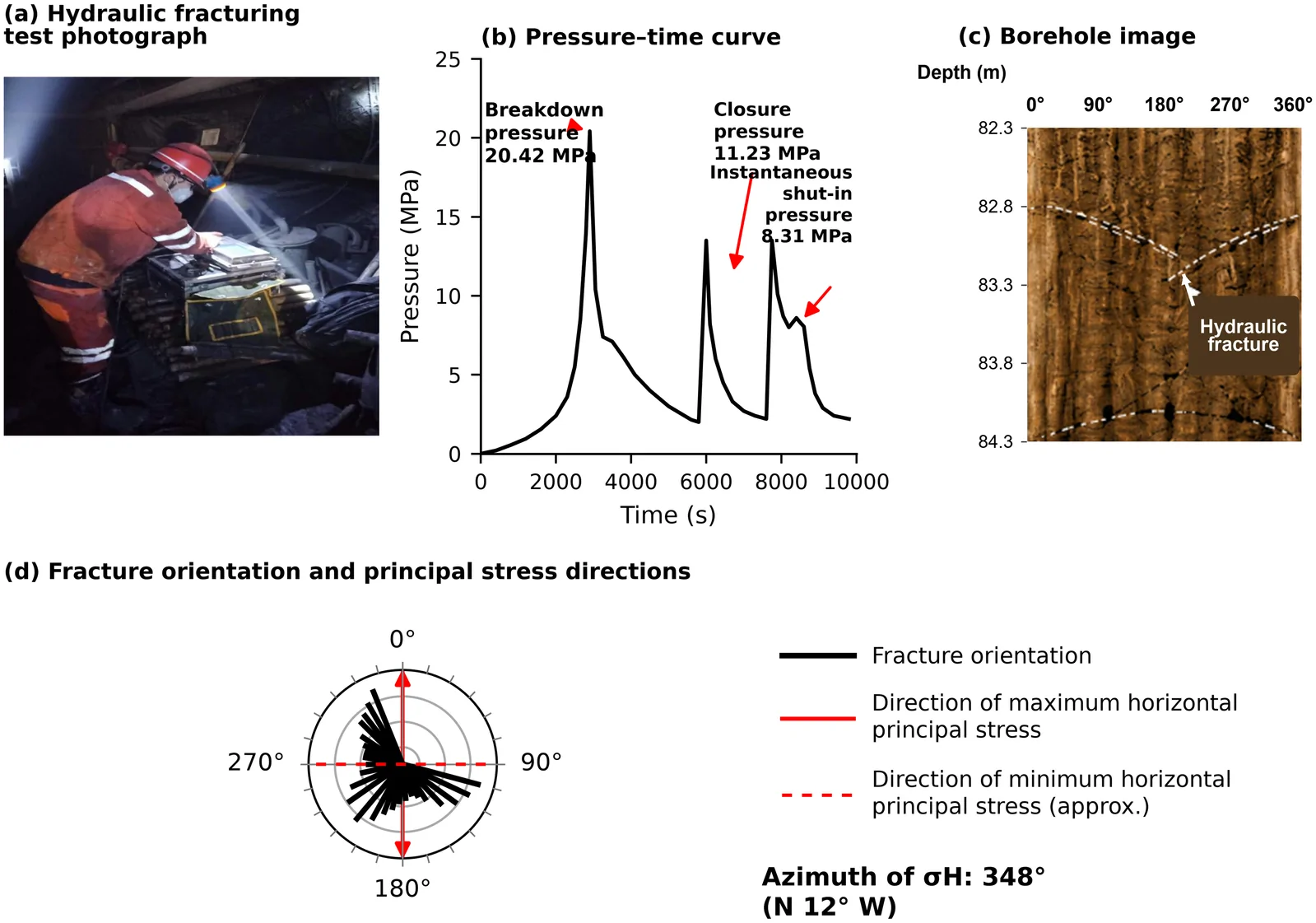
In-situ stress testing and fracture orientation identification. All panels were created from the authors’ field measurements, analyses, and photographs taken by the research team during onsite testing; no third-party content was reproduced.

### 5.2 Staged fracturing operations and parameter response

Well HYL1 was subjected to six hydraulic-fracturing stages. Pumping rate, injected-fluid volume, breakdown pressure, and shut-in pressure are compared in Table 4. Across the six stages, the pumping rate was 5.0–9.0 m^3^/min, the injected-fluid volume was 292.98–562.64 m^3^, the breakdown pressure was 7.14–17.01 MPa, and the shut-in pressure was 2.74–8.51 MPa. All stages completed the designed fluid-injection and proppant-placement programs without major well leakage, pump sticking, or sustained abnormal pressure surges, supporting the field implementability of the near-roof scheme. The stage-resolved construction and pressure curves are shown in Fig 12.

**Table 4:** Summary of hydraulic-fracturing parameters for the six stages in Well HYL1.

Stage	Pumping rate (m^3^/min)	Injected-fluid volume (m^3^)	Breakdown pressure (MPa)	Shut-in pressure (MPa)
1	5.0	292.98	13.60	6.06
2	8.0	498.25	11.90	7.36
3	8.5	477.06	15.02	7.11
4	8.7	473.15	11.00	2.74
5	8.8	562.64	17.01	8.51
6	9.0	486.59	7.14	6.84

**Fig 12:**
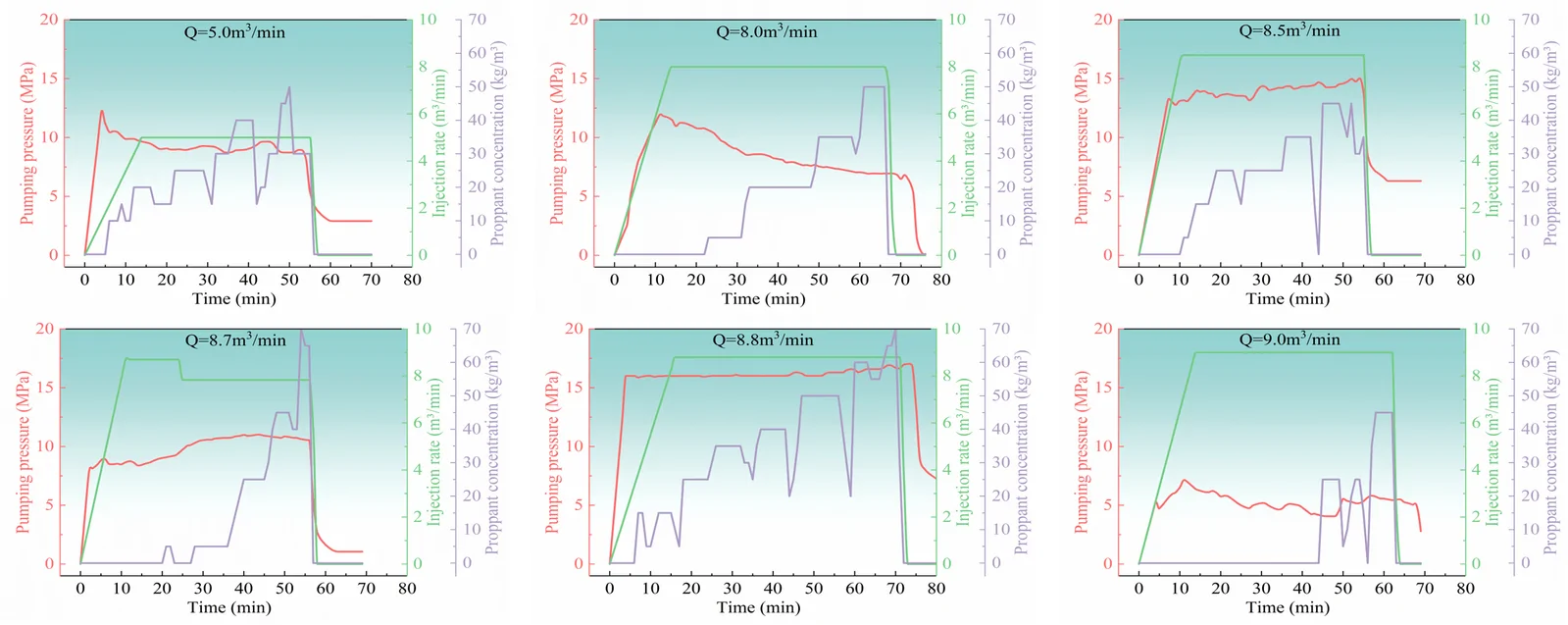
Construction-response curves for the six hydraulic-fracturing stages in Well HYL1.

The first stage used a pumping rate of 5.0 m^3^/min and reached a breakdown pressure of 13.60 MPa. Stages 2–6 used similar pumping rates of 8.0–9.0 m^3^/min, yet their breakdown pressures ranged from 7.14 to 17.01 MPa. The relatively high pressures in Stages 3 and 5 (15.02 and 17.01 MPa) despite comparable pumping rates show that pumping rate alone did not determine fracture initiation. The stage-to-stage variation may reflect local lithology, natural-fracture development, near-wellbore friction, perforation conditions, interlayer stress differences, and stress interference from preceding stages. A higher pumping rate can improve fluid supply during pressure accumulation and propagation, but its influence cannot be separated from these interval-specific conditions in the field data.

Accordingly, the six field stages are not single-variable controlled experiments. The nonmonotonic breakdown pressures in Table 4 should be interpreted as interval-specific responses under combined geological and operational controls, rather than as a direct rate–pressure relationship.

### 5.3 Gas drainage response and fracturing effectiveness evaluation

Post-fracturing monitoring showed that the mixed-gas drainage rate generally remained between 630 and 690 m^3^/d, the pure-methane drainage rate was approximately 520–570 m^3^/d, and methane concentration remained above 82%. Meanwhile, flowing pressure decreased from approximately 530–290 kPa, a cumulative reduction of about 45.3%. The stable methane concentration and drainage rates, together with the declining flowing pressure, indicate persistent gas supply and hydraulic-fracture connectivity under the monitored operating conditions. The corresponding time-series responses are shown in Fig 13.

**Fig 13:**
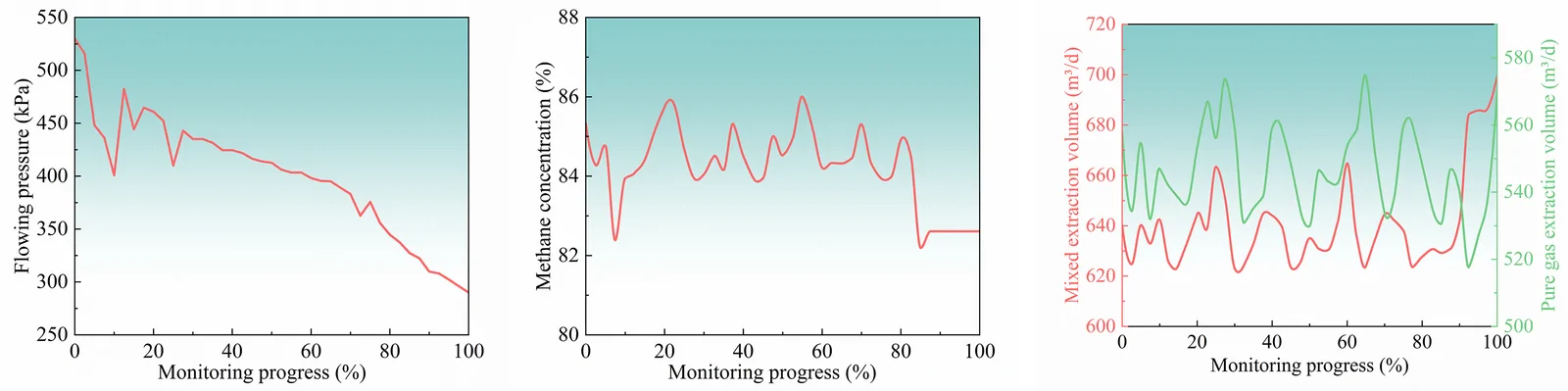
Time-series responses of post-fracturing gas-drainage parameters.

During the middle and late mining stages, both mixed-gas and pure-methane drainage rates increased. This pattern may indicate that mining-induced stress changes reopened some hydraulic fractures and connected them with natural and mining-induced fractures. The continued decline in flowing pressure, together with stable drainage rates, is consistent with sustained gas supply and flow connectivity in the fractured zone.

The mixed-gas drainage rate, methane concentration, and pure-methane drainage rate are not independent indicators, because the pure-methane rate is calculated from the mixed-gas rate and methane concentration. Moreover, no direct pre- and post-fracturing permeability measurements or untreated control interval were available. The field results are therefore interpreted as evidence of sustained fracture connectivity and improved gas-migration conditions, rather than as a direct quantitative measurement of permeability enhancement.

### 5.4 Multiscale consistency and engineering applicability

Laboratory experiments, numerical simulations, and field observations show qualitative consistency in the proposed mechanism, but their injection rates are not directly scaled. The laboratory tests show that stronger confinement raises breakdown and stable propagation pressures and shifts failure from dispersed tensile cracking toward localized mixed-mode damage. The numerical model resolves the associated coupling among pore pressure, effective stress, damage, and displacement. The field trial demonstrates constructability and provides drainage evidence consistent with sustained fracture connectivity and improved gas-migration conditions. These cross-scale results support mechanistic consistency rather than a direct quantitative scaling law.

Future work could deepen the monitoring and mechanism comparison in several directions. Waveform-level Hilbert-Huang transform analysis has shown that the frequency-energy evolution of AE signals can distinguish loading and instability stages in coal-rock materials [49]. At the field scale, studies of structural-surface-controlled coal-rock failure and integrated microseismic-electromagnetic-radiation monitoring provide complementary approaches for tracking instability processes [50,51]. Alternative stimulation research has further shown that liquid-nitrogen cold soaking can reorganize coal pore structure and fractal characteristics [52], offering a useful contrast with the fracture-network connectivity generated by hydraulic stimulation. More generally, RA-AF analysis has also been applied to tensile-shear crack classification in imitation steel-fiber concrete [53], indicating the broader utility of these AE descriptors while reinforcing the need for material- and system-specific interpretation thresholds.

## 6 Conclusion and outlook

(1) Near-roof hydraulic fracturing enhances coal-seam permeability through the coupled effects of pore-pressure accumulation, effective-stress reduction, roof damage, and cross-layer fracture connection. Damage-induced stiffness degradation redistributes the local load in the roof, while fractures crossing the coal–rock interface connect roof fractures with coal-seam cleats and provide additional pathways for methane migration. Cross-layer fractures further connect coalbed cleats and natural fractures, expanding the effective flow cross-section and reducing gas migration resistance, thereby achieving coordinated pressure relief in the roof and enhanced permeability in the coal seam.
(2) The injection rate governs the dynamic balance among fluid inflow, matrix filtration loss, and near-wellbore pore pressure accumulation. As the injection rate increases from 0.1 to 0.4 mL/s, the peak pressure increases from 4.30 to 6.79 MPa, whereas the stable propagation pressure remains within 3.91–4.94 MPa. This response suggests that a higher injection rate provides a greater driving force for fracture initiation and accelerates near-wellbore pressure buildup. After the fracture enters the stable propagation stage, however, its subsequent growth is controlled primarily by the coupled effects of fracture resistance, confining stress constraints, filtration characteristics, and the coal–rock interface.
(3) Triaxial in-situ stress governs the dominant fracture propagation direction, failure mode, and degree of damage localization. As the stress combination increases from 3/4/6 MPa to 9/12/18 MPa, the breakdown pressure rises from 5.15 MPa to 12.23 MPa, accompanied by enhanced closure of natural fractures and increased normal confinement on fracture surfaces. The pressure-acoustic emission (AE) response gradually transitions from dispersed tensile damage to phased, localized tensile-shear composite failure. Numerical results further reveal that the minimum principal stress controls the normal orientation of fracture planes, while the intermediate principal stress and principal stress difference regulate pore pressure diffusion, the width of the SDEG damage zone, and local displacement responses.
(4) Following six-stage near-roof hydraulic fracturing of Well HYL1, the mixed-gas drainage rate remained at 630–690 m^3^/d and methane concentration exceeded 82%. Together with the decline in flowing pressure, these data support the engineering feasibility of the staged near-roof scheme and indicate sustained fracture connectivity and favorable gas-migration conditions; they do not constitute a direct permeability measurement. Future work should quantify fracture geometry and permeability by combining natural coal–rock assemblages, three-dimensional AE localization, computed-tomography reconstruction, microseismic monitoring, and long-term multi-well controls.

## Supporting information

S1 Data Source data for the laboratory hydraulic-fracturing tests, acoustic-emission analyses, in-situ-stress measurements, six-stage field-fracturing comparison, and post-fracturing gas-drainage monitoring. (ZIP)

S2 Data Abaqus model and input files for the hydro-mechanically coupled numerical simulations. (ZIP)
